# Role of hydrogen sulfide in sulfur dioxide production and vascular regulation

**DOI:** 10.1371/journal.pone.0264891

**Published:** 2022-03-17

**Authors:** Chufan Sun, Wen Yu, Boyang lv, Yanan Zhang, Shuxu Du, Heng Zhang, Junbao Du, Hongfang Jin, Yan Sun, Yaqian Huang

**Affiliations:** 1 Department of Pediatrics, Peking University First Hospital, Beijing, China; 2 Department of Cardiology, Beijing Children’s Hospital, Capital Medical University, National Center for Children’s Health, Beijing, China; 3 Department of Pediatrics, Beijing Shijitan Hospital, Capital Medical University, Beijing, China; 4 Department of Endocrinology, Beijing Chaoyang Hospital, Capital Medical University, Beijing, China; 5 Key Laboratory of Molecular Cardiovascular Sciences, Ministry of Education, Beijing, China; University Medical Center Utrecht, NETHERLANDS

## Abstract

Both hydrogen sulfide (H_2_S) and sulfur dioxide (SO_2_) are produced endogenously from the mammalian metabolic pathway of sulfur-containing amino acids and play important roles in several vascular diseases. However, their interaction during the control of vascular function has not been fully clear. Here, we investigated the potential role of H_2_S in SO_2_ production and vascular regulation *in vivo* and *in vitro*. Wistar rats were divided into the vehicle, SO_2_, DL-propargylglycine (PPG) + SO_2_, β-cyano-L-alanine (BCA) + SO_2_ and sodium hydrosulfide (NaHS) + SO_2_ groups. SO_2_ donor was administered with or without pre-administration of PPG, BCA or NaHS for 30 min after blood pressure was stabilized for 1 h, and then, the change in blood pressure was detected by catheterization via the common carotid artery. Rat plasma SO_2_ and H_2_S concentrations were measured by high performance liquid chromatography and sensitive sulfur electrode, respectively. The isolated aortic rings were prepared for the measurement of changes in vasorelaxation stimulated by SO_2_ after PPG, BCA or NaHS pre-incubation. Results showed that the intravenous injection of SO_2_ donors caused transient hypotension in rats compared with vehicle group. After PPG or BCA pretreatment, the plasma H_2_S content decreased but the SO_2_ content increased markedly, and the hypotensive effect of SO_2_ was significantly enhanced. Conversely, NaHS pretreatment upregulated the plasma H_2_S content but reduced SO_2_ content, and attenuated the hypotensive effect of SO_2_. After PPG or BCA pre-incubation, the vasorelaxation response to SO_2_ was enhanced significantly. While NaHS pre-administration weakened the SO_2_-induced relaxation in aortic rings. In conclusion, our *in vivo* and *in vitro* data indicate that H_2_S negatively controls the plasma content of SO_2_ and the vasorelaxant effect under physiological conditions.

## Introduction

Sulfur dioxide (SO_2_) was previously considered a toxic gas, but it has been proven that it can be endogenously produced from the metabolism of sulfur-containing amino acids, with L-cysteine as a substrate and catalyzed by aspartate aminotransferase (AAT) [1,2]. Our previous studies revealed the existence of the SO_2_/AAT pathway in arteries and its vasodilator function [3]. Studies have also confirmed its effect on vascular function [4]. Supplementation with SO_2_ donors could protect against various cardiovascular diseases. Emerging evidence indicated that endogenous SO_2_ is a new gasotransmitter involved in cardiovascular regulation. Interestingly, in a variety of pathological models, including atherosclerosis [5], pulmonary hypertension [6], and myocardial ischemia-reperfusion injury [7], SO_2_ was found to affect the production of another gasotransmitter, hydrogen sulfide (H_2_S). However, the interaction between H_2_S and SO_2_ under physiological conditions is largely unknown.

L-Cysteine is used as a substrate to produce the endogenous H_2_S in the cardiovascular system through cystathionine-γ-lyase (CSE) [8–11]. Endogenous H_2_S not only has physiological functions, such as vasorelaxation, but also has pathophysiological effects, including the inhibition of hypertension [10,12–17]. Recently, we showed that in a monocrotaline-induced pulmonary hypertensive rat model, the H_2_S pathway in pulmonary artery endothelial cells is damaged, which reduces the sulfhydration of AAT, thus enhancing AAT activity and increasing SO_2_ production [18]. However, it still unclear whether H_2_S affects the SO_2_ pathway under physiological conditions.

As mentioned above, the importance of H_2_S and SO_2_ for the modulation of blood pressure and arterial tension has been gradually revealed, but their interaction in the control of blood pressure and vascular function has not been clear. Thus, our research aimed at exploring the possible role of H_2_S in SO_2_ production and vascular regulation under physiological conditions.

## Materials and methods

### Reagents

Acetylcholine chloride (ACH) and phenylephrine (PE) were from Beijing Chemical Reagent Company and Tianjin Amino Acid Company in China, respectively. Sodium sulfite and sodium bisulfite (Na_2_SO_3_/NaHSO_3_, the SO_2_ donor), DL-propargylglycine (PPG, a selective CSE inhibitor), β-cyano-L-alanine (BCA, another CSE inhibitor), and sodium hydrosulfide (NaHS, the H_2_S donor) were from Sigma, USA. Since SO_2_, HSO_3_^–^ and HSO_3_^2–^ can be transformed into each other in biological system [1], NaHSO_3_/Na_2_SO_3_ was used as the SO_2_ donor (S1 Fig). NaHSO_3_ and Na_2_SO_3_ were dissolved in deionized water at a molar ratio of 1:3. The fresh SO_2_ donor stock solution was then diluted with bath solution to obtain a series of working solutions with different concentrations. The handling and properties of NaHS were given in a brochure by Stauffer (1974). It is produced by the absorption of H_2_S in sodium hydroxide and shipped as a 45% solution with a specific gravity of 1.303 and a pH of 10.4 [19]. NaHS powder was rapidly dissolved in normal saline (0.9%) to obtain the desired concentration of stock solutions (pH 7.4) which was immediately injected intravenously into the right external iliac vein of rats or added into the organ bath solution of aortic rings (37°C). Previous study showed that given a physiological pH around 7.4 and temperature of 37°C, NaHS solution will yield about one-third of the undissociated H_2_S gas and the other two-thirds remain as HS^−^[20]. The composition of the Krebs’ solution with pH 7.2–7.4 was as follows: NaCl (120 mmol/L), KCl (5.5 mmol/L), NaHCO_3_ (20 mmol/L), CaCl_2_ (2.5 mmol/L), MgCl_2_·6H_2_O (1.2 mmol/L), NaH_2_PO_4_ (1.2 mmol/L), EDTA-Na_2_ (0.03 mmol/L) and glucose (10 mmol/L).

### Animal experiment

Animal care and operation procedures were carried out strictly in accordance with the Animal Management Rule of the Ministry of Health of the People’s Republic of China (Documentation 55, 2001). The protocol was approved by the Animal Care Committee of Peking University First Hospital (Protocol Number: J202044) and conformed to the ARRIVE guidelines (https://journals.plos.org/plosbiology/article?id=10.1371/journal.pbio.3000411). Forty male Wistar rats (body weight, 200±5 g) were purchased from the Experimental Animal Center, Peking University Health Science Center (Beijing, China). They were housed at a constant temperature of 25°C under a 12-h light-dark cycle and maintained on *ad libitum* food and water. Rats were monitored twice daily for health status and husbandry conditions. In the health monitoring, we closely observed the food consumption, water intake, body weight and general assessment of rat activity, panting, and fur condition of the rats. During the whole experiment, intraperitoneal injection of sodium pentobarbital (45 mg/kg) was used for anesthesia and supplemented with an additional dose of 10 mg/kg. All efforts were made to minimize suffering. They were randomly assigned to five groups (n = 8 each) as follows: (1) vehicle group where the rats were intravenously injected with the equal volume of physiological saline; (2) SO_2_ group where the rats were intravenously injected with Na_2_SO_3_/NaHSO_3_ (40 μmol/kg, pH 7.4); (3) PPG + SO_2_ group where the rats were given PPG (30 mg/kg) intravenously and then Na_2_SO_3_/NaHSO_3_ (40 μmol/kg) 30 min later; (4) BCA + SO_2_ group where the rats were given BCA (50 mg/kg) intravenously and then Na_2_SO_3_/NaHSO_3_ (40 μmol/kg) 30 min later; (5) NaHS + SO_2_ group where the rats were given NaHS (56 μmol/kg) [21,22] intravenously and then Na_2_SO_3_/NaHSO_3_ (40 μmol/kg) [23,24] 30 min later. PPG (30 mg/ml), BCA (50 mg/ml), NaHS (56 μmol/ml) or Na_2_SO_3_/NaHSO_3_ (40 μmol/ml) in a final volume of 200 μl was injected intravenously into the femoral vein within a minute. After the experiment, the rats were euthanized with intravenous injection of an overdose of sodium pentobarbital.

### Preparation of rat model and measurement of blood pressure

Two polyethylene catheters were inserted into the left common carotid artery (LCCA) and the right external iliac vein. The mean blood pressure (MBP) was measured via the LCCA, and the extracorporeal end of the catheter was connected to a pressure sensor and PowerLab Software for recording blood pressure and respiratory conditions. (BL-410, Chengdu TME Technology, China) [25]. The extracorporeal end of catheter in the right external iliac vein was connected to a 5 ml syringe for intravenous bolus injection of chemicals and collection of venous blood samples.

### Measurement of plasma H_2_S and SO_2_ content

Plasma sulfide levels were detected using a free radical analyzer TBR4100 with an H_2_S-selective sensor (World Precision Instruments, China) to reflect H_2_S content as previously described [26]. Briefly, the electrode was activated in deionized water for more than 2 h. The measured item of this analyzer was set to millivolt. The sensitive sulfur electrode was immersed into 1 mL of sample, so was the reference electrode. And the millivolt value was recorded after the reading was stable. Plasma SO_2_ concentration was detected by HPLC [3]. Briefly, the sulfite in the sample was reduced to a sulfhydryl compound by the addition of sodium borohydride. Then, it was combined with monobromobimane, and perchloric acid was added to remove the protein in the sample, which was neutralized by use of Tris-HCl (pH 3.0). Small sulfhydryl molecules were separated from other fractions through chromatographic analysis and determined with a fluorescence detector.

### AAT activity assay

Purified AAT protein (Roche Diagnostics, Mannheim, Germany) was incubated with or without PPG (100 μmol/L) for 30 min at 37°C in PBS buffer. After incubation, the activity of AAT was measured using AAT Assay Kit (Jiancheng Bioengineering Institute, Nanjing, China) according to the manufacturer’s instructions and was expressed in Carmen’s Unit.

### Preparation of rat aortic rings

Thoracic aortas were rapidly isolated from anesthetized male Wistar rats (n = 8 per group) with the removal of adherent adipose and connective tissue. Each aorta was cut into 3–4 aortic rings of 3 mm in length and immersed in Krebs’ buffer at 4°C. During the whole process, artificially overstretching blood vessels was avoided to maintain their activity.

### Measurement of the rat aortic contractility

The aortic rings were immersed into organ baths containing oxygenated Krebs’ buffer at 37.5°C, fixed, and connected with a tension sensor. The latter was then connected with a multi-channel physiological recorder to record the tension value and display them through PowerLab software [3]. The rings were first stretched to a tension of 1 g (international unit 9.8×10^−3^ Newton) and equilibrated for 60 min. During this period, the incubation solution was replaced every 15 min. After the tension stabilized, the aortic rings were ready for testing.

To confirm the vascular reactivity, the aortic rings were first treated with PE (1 μmol/L) for contraction. After reaching equilibrium, they were treated with ACH (1 μmol/L) for relaxation. Those with good reactivity were used for the following procedures. After the peak of relaxation, the rings were rinsed with Krebs’ buffer three times. When the tension of the rings became stable again and returned to the initial level, the aortic rings were incubated with vehicle, PPG (100 μmol/L), BCA (100 μmol/L) or NaHS (50 μmol/L) for 10 min, and then PE (3 μmol/L) was added to make the rings in precontraction state. After that, a dose-response to Na_2_SO_3_/NaHSO_3_ (50–1000 μmol/L) in aortic rings was detected. Relaxation was expressed as the percentage reduction of the maximum precontraction achieved by PE.

In the experiment of measuring vasoconstriction response, the rings were first stretched to a tension of 1 g, equilibrated for 20 min, and then contracted with 60 mmol/L KCl for 15 min to test its contractility. After rinsing with Krebs’ buffer three times and the tension stabilized, the rings were incubated with Na_2_SO_3_/NaHSO_3_ (1 mmol/L) or PPG (100 μmol/L) plus Na_2_SO_3_/NaHSO_3_, and subsequently a dose-response to PE was detected. Contraction was expressed as the percentage of its peak contraction with 60 mmol/L KCl.

### Measurement of plasma nitric oxide (NO) content

The plasma NO content was detected using the NO assay kit (Applygen, Beijing, China). Briefly, 50 μL of standard and plasma sample were added to a 96-well plate and 50 μl of Griess R1 solution was then added to these wells and incubated at 37°C for 2 h. After that, 50 μl of Griess R2 solution was added and incubated in dark at 37°C for 5 min, and the absorbance values were measured at 540 nm.

### Western blot

The aortic tissues were homogenated with lysis buffer containing protease inhibitors and phosphatase inhibitors. Then, the supernatants were obtained by centrifuging at 12000 g for 20 min. An equal amount of aortic protein was isolated by SDS-PAG electrophoresis and transferred to nitrocellulose (NC) membranes. The NC membranes were blocked using 5% skim milk and then incubated with heme oxygenase (HO)-1 antibody (diluted 1:1000, Enzo Life Sciences, Farmingdale, NY, USA), HO-2 antibody (diluted 1:1000, Enzo Life Sciences), endothelial NO synthase (eNOS) antibody (diluted 1:1000, Cell Signaling Technology, Danvers, MA, USA), and β-tubulin (diluted 1:5000, Beyotime Biotechnology, Beijing, China) overnight, respectively. After that, the membranes were incubated with horseradish peroxidase-conjugated secondary antibodies (Sigma-Aldrich Corporation, St Louis, MO, USA). The bands were detected with a chemiluminescence detection system (ProteinSimple, San Francisco, CA, USA).

### Statistics

SPSS 15.0 (SPSS, Chicago, IL, USA) was used for statistical analysis. Data are expressed as the mean±SEM. A Student’s t-test was performed to compare differences between two groups. Paired-sample t-test was performed to compare the changes of plasma H_2_S and SO_2_ contents in the rats before and after treatment. One-way ANOVA followed by least significant difference (LSD) test was performed to compare differences among multiple groups. P values less than 0.05 were considered significant.

## Results

### H_2_S inhibits the plasma levels of SO_2_ *in vivo*

**Fig 1:**
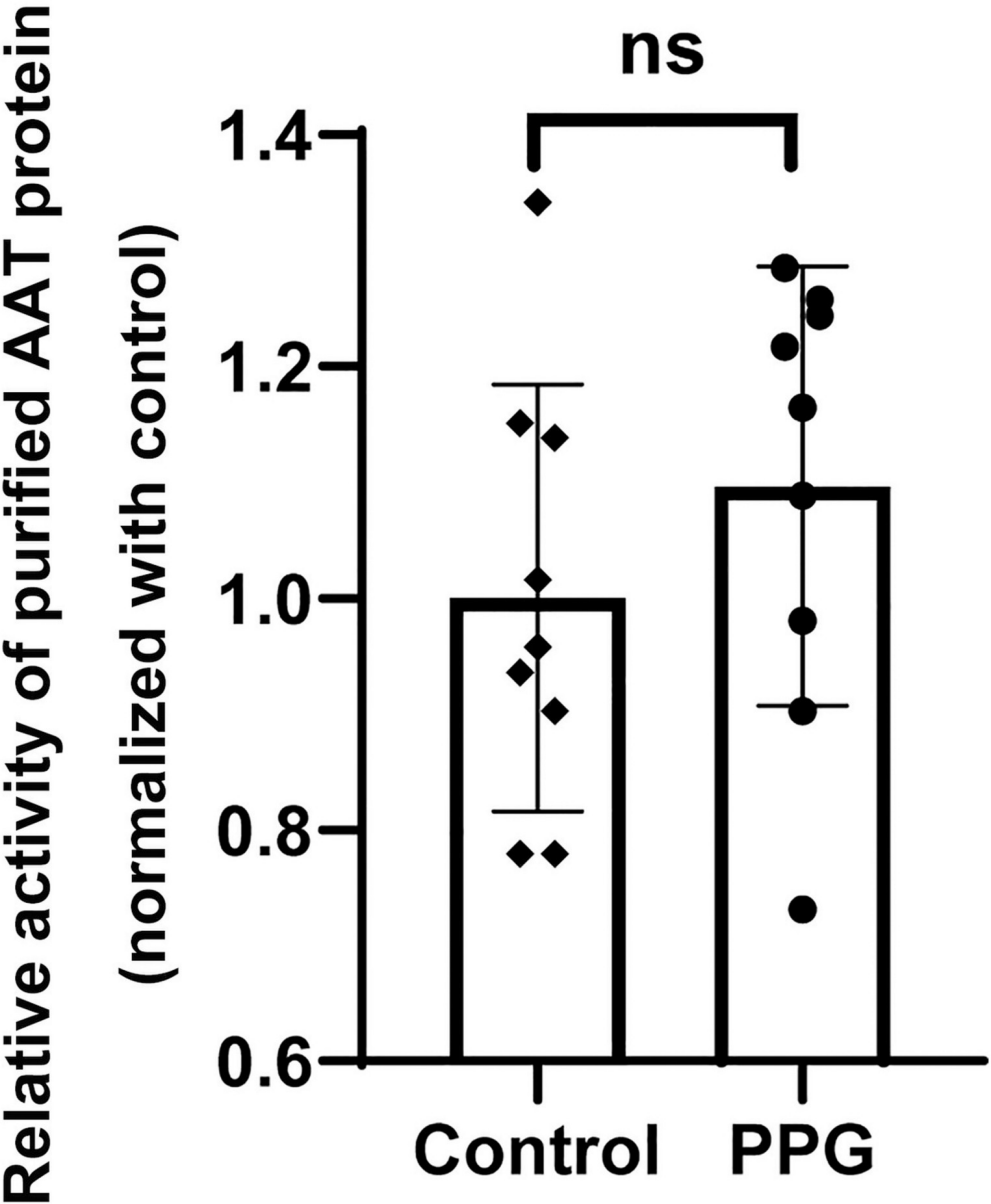
PPG had no direct effect on AAT activity. Purified AAT protein was incubated with or without PPG (100 μmol/L) for 30 min at 37°C. Data are presented as mean ± SEM; n = 9 in each group; Student’s t-test; ns, not significant.

To investigate the effect of H_2_S on SO_2_ level *in vivo*, the rats were intravenously injected with PPG, BCA or NaHS to downregulate or upregulate H_2_S level. As shown in Table 1, after intravenous injection of PPG (30 mg/kg), the rat plasma H_2_S concentration was decreased significantly (from 20.22±0.31 μmol/L to 11.65±0.47 μmol/L), whereas the plasma SO_2_ content was increased notably (from 15.33±0.72 μmol/L to 21.55±2.41 μmol/L). In rats injected with BCA, another CSE inhibitor, we observed similar findings as in PPG-treated rats. Conversely, rats injected with NaHS to increase plasma H_2_S content (from 20.60±0.40 μmol/L to 34.75±1.47 μmol/L) exhibited a reduced plasma SO_2_ content (from 15.16±0.65 μmol/L to 12.75±1.12 μmol/L). To explore whether PPG had any direct effect on SO_2_ production, the purified SO_2_ synthase AAT protein was treated with or without PPG in PBS buffer. The results showed that there was no difference in the activity of the purified AAT protein between the control group and the PPG group (Fig 1), implying that PPG had no direct effect on the SO_2_ production. These data suggest that H_2_S negatively regulates the plasma levels of SO_2_.

**Table 1:** Plasma contents of H_2_S and SO_2_ in rats before and after treatment with PPG, BCA or NaHS.

Groups	H_2_S content (μmol/L)			SO_2_ content (μmol/L)	
	0 min	5 min	30 min	0 min	30 min
**PPG**	20.22±0.31	–	11.65±0.47*	15.33±0.72	21.55±2.41^#^
**BCA**	20.63±0.34	–	11.00±0.83*	12.48±0.30	20.58±1.52^#^
**NaHS**	20.60±0.40	34.75±1.47*	20.94±0.31	15.16±0.65	12.75±1.12^#^

Data expressed as mean ± SEM; n = 8 in each group; paired-sample t-test

*P<0.05 compared with the plasma H_2_S content of rats before treatment in the corresponding group

^#^P<0.05 compared with the plasma SO_2_ content of rats before treatment in the corresponding group. PPG, DL-propargylglycine; BCA, β-cyano-L-alanine; NaHS, sodium hydrosulfide.

### H_2_S attenuated the hypotensive effect of SO_2_ *in vivo*

**Fig 2:**
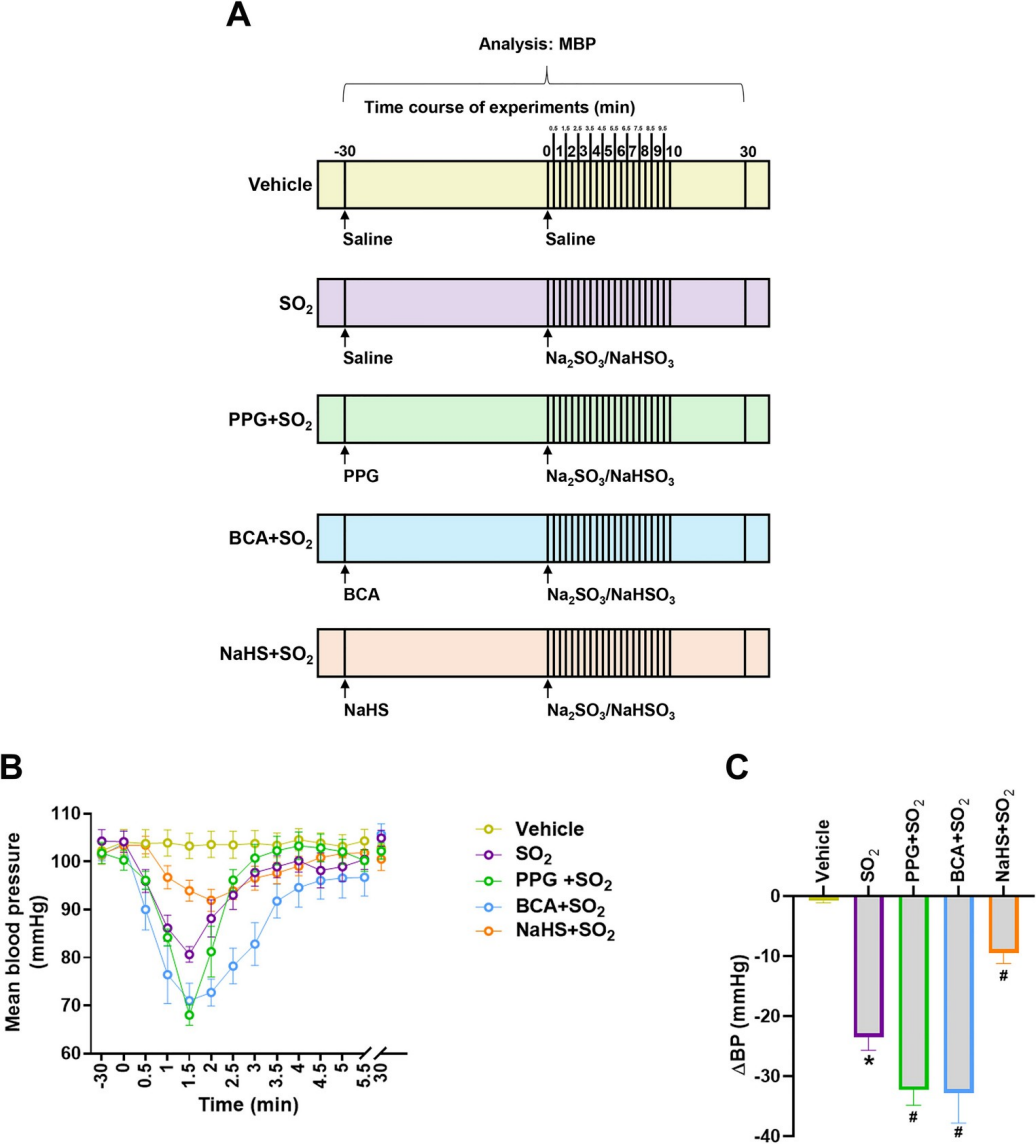
H_2_S inhibited the hypotension induced by SO_2_ *in vivo*. (A) Schematic diagram of strategies to explore the influence of the interaction of H_2_S and SO_2_ on blood pressure regulation. Rats were intravenously injected with PPG (30 mg/kg), BCA (50 mg/kg), NaHS (56 μmol/kg) or equal volume of physiological saline. After 30 minutes, the rats were given an intravenous injection of Na_2_SO_3_/NaHSO_3_ (40 μmol/kg) or equal volume of saline, and then their blood pressure was continuously monitored for 30 minutes. Mean blood pressure (MBP) of rats was analyzed at different time points. (B) Changes in MBP of rats in each group at different time points. Data are mean±SEM; n = 8 in each group. (C) Comparison of the drop in MBP of rats in each group after administration of Na_2_SO_3_/NaHSO_3_ for 1.5 minutes. Data are presented as mean±SEM; n = 8 in each group; One-way ANOVA followed by LSD post hoc test; *P<0.05 compared with vehicle group; ^#^P<0.05 compared with SO_2_ group.

To explore the significance of SO_2_ level inhibition by H_2_S, the rats were intravenously injected with physiological saline, PPG, BCA or NaHS for 30 minutes, followed by SO_2_ derivatives (Na_2_SO_3_/NaHSO_3_) injection, and the changes of their blood pressure were monitored in real time (Fig 2A). Injection of physiological saline did not affect MBP of rats in vehicle group (Fig 2B). However, the MBP of rats in the SO_2_ group dropped by 22.55% within 1.5 minutes after the intravenous injection of SO_2_ derivatives; then, the MBP began to rise, and returned to the basal level 10 minutes after injection of SO_2_ derivatives (Fig 2B). The rat MBP in the SO_2_ group was significantly lower than that in the vehicle group within 1.5 minutes after the injection of SO_2_ derivatives (Fig 2C). In the PPG+SO_2_ group, there was no significant difference in MBP between the baseline and 30 minutes after administration of PPG; the MBP dropped by 32.19% within 1.5 min after SO_2_ derivatives injection; then it began to rise, and returned to baseline 10 minutes after SO_2_ derivatives injection (Fig 2B). The decrease in MBP of the PPG+SO_2_ group was markedly greater than that of the SO_2_ group within 1.5 minutes after SO_2_ derivatives injection (Fig 2C). Similarly, the administration of rats with BCA, another CSE inhibitor, also exacerbated the hypotensive effect of SO_2_. The MBP was significantly decreased within 1 minute after H_2_S donor NaHS injection (S2 Fig) and then returned to the basal level within 30 minutes (Fig 2B). Although pretreatment of rats with NaHS caused a decrease in MBP within 1–3 minutes after the injection of SO_2_ derivatives, the decrease in MBP was significantly smaller than that of the SO_2_ group (Fig 2). These data suggest that H_2_S could inhibit the hypotensive effect of SO_2_.

### H_2_S negatively controls the vasorelaxant effect of SO_2_ *in vitro*

**Fig 3:**
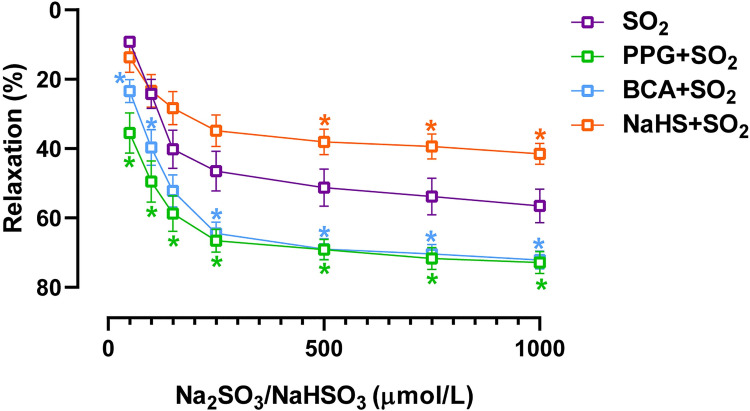
H_2_S inhibited the vasorelaxation induced by SO_2_ *in vitro*. Rat aortic rings were incubated with vehicle, PPG (100 μmol/L), BCA (100 μmol/L) or NaHS (50 μmol/L) for 10 minutes, then precontracted with PE (3 μmol/L), and finally treated with various concentrations of Na_2_SO_3_/NaHSO_3_ (50–1000 μmol/L). Data are presented as mean±SEM; n = 8 in the SO_2_ group, PPG+SO_2_ group and NaHS+SO_2_ group, and n = 10 in the BCA+SO_2_ group; One-way ANOVA followed by LSD post hoc test; *P<0.05 compared with SO_2_ group.

**Fig 4:**
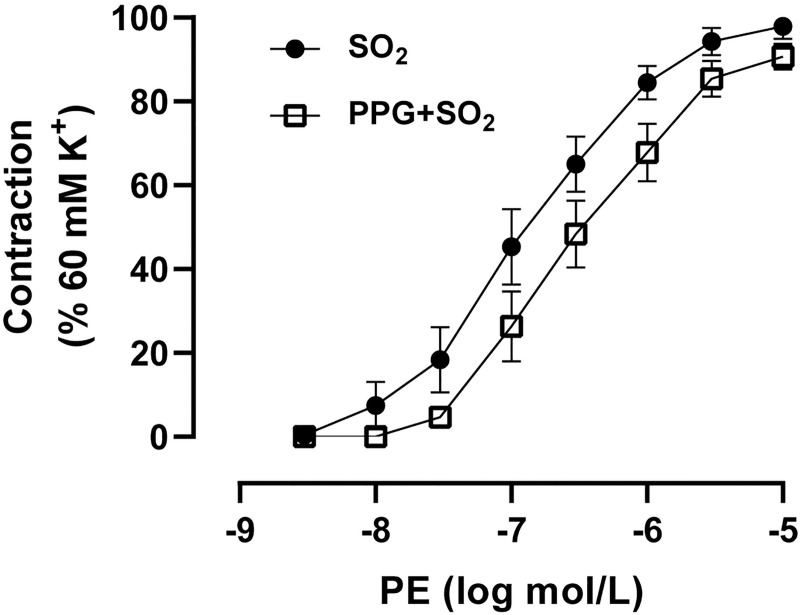
H_2_S did not influence the effect of SO_2_ on the phenylephrine (PE)-elicited vasoconstriction. Rat aortic rings were incubated with Na_2_SO_3_/NaHSO_3_ (1 mmol/L) or PPG (100 μmol/L) plus Na_2_SO_3_/NaHSO_3_ (1 mmol/L), and then treated with various concentrations of PE (0.003–10 μmol/L). Data are presented as mean±SEM; n = 8 in each group; Student’s t-test.

Vasorelaxation of arteries could result in a reduction in blood pressure. SO_2_ derivatives (50 to 1000 μM) exerted a concentration-dependent dilatory effect in the aortic rings precontracted with 3 μmol/L PE (Fig 3). SO_2_ derivatives-elicited vasodilation was significantly increased in the aortic rings preincubated with either PPG or BCA but decreased in aortic rings pretreated with NaHS (Fig 3). In addition, there was no significant difference in PE-elicited constriction in aortic rings between SO_2_ group and PPG+SO_2_ group (Fig 4). These results demonstrate that H_2_S might inhibit the decrease of vascular tone caused by SO_2_ through weakening the SO_2_-induced vasodilation, thus attenuating the transient hypotensive effect of SO_2_.

### NO/eNOS and CO/HO pathway did not change during the regulation of H_2_S on SO_2_

**Fig 5:**
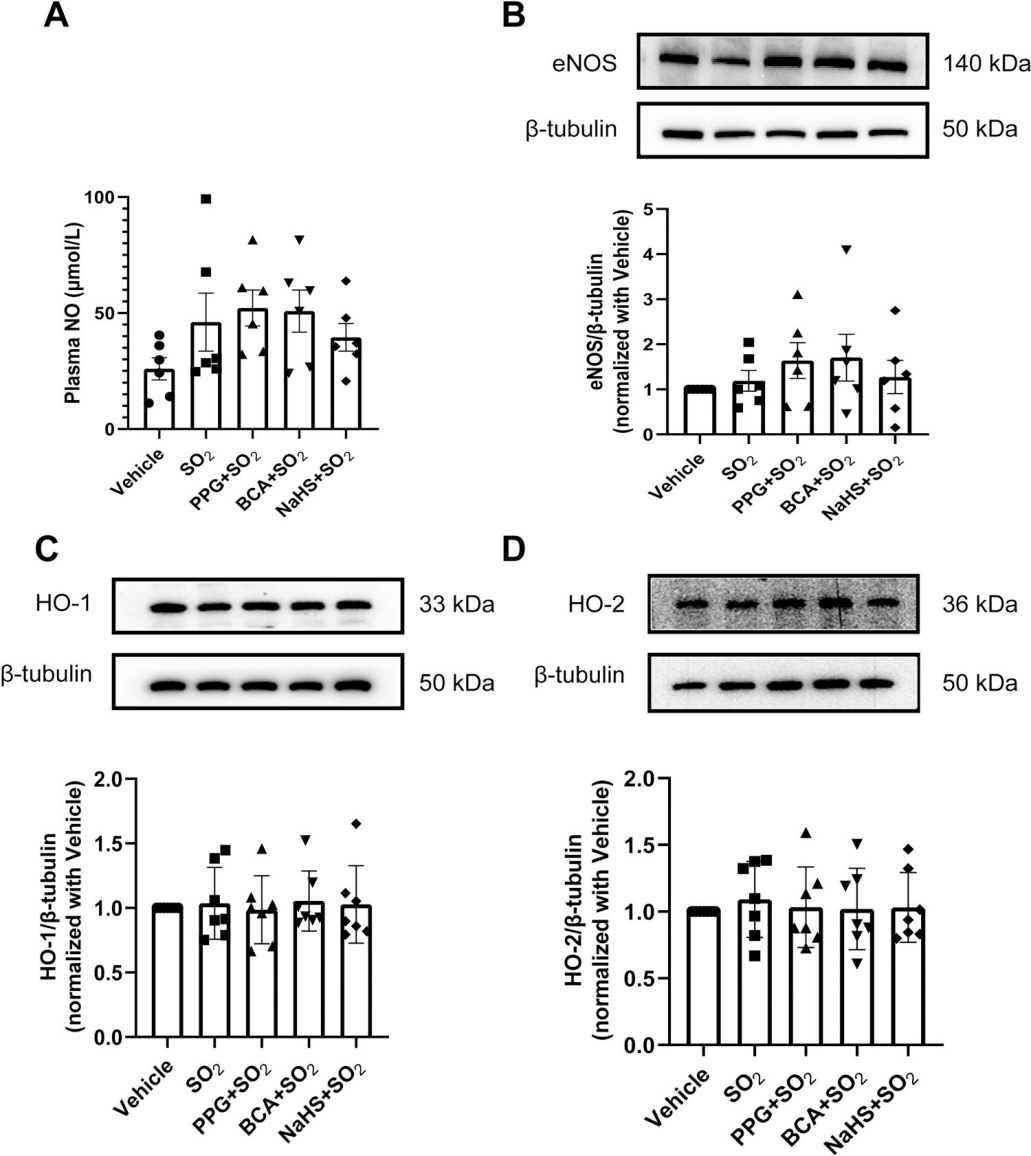
NO/eNOS and CO/HO pathway did not change during the regulation of H_2_S on SO_2_. Rats were intravenously injected with PPG (30 mg/kg), BCA (50 mg/kg), NaHS (56 μmol/kg) or equal volume of physiological saline. After 30 minutes, the rats were given an intravenous injection of Na_2_SO_3_/NaHSO_3_ (40 μmol/kg). Blood samples and aortic tissues were collected after 1.5 minutes. (A) Plasma NO content in each group. (B-D) Representative western blots and quantitative analysis of eNOS (B), HO-1 (C) and HO-2 (D) in the aortic tissues of each group. Data are presented as mean±SEM; n = 6–7 in each group; One-way ANOVA followed by LSD post hoc test.

To investigate whether NO and CO are involved in the regulation of H_2_S on SO_2_ action, we detected the plasma NO content and the expressions of NO producing enzyme eNOS and CO producing enzymes HO-1 and HO-2 in the rat aortic tissues. The data showed that the plasma NO content (Fig 5A) and the protein expressions of aortic eNOS (Fig 5B), HO-1 (Fig 5C) and HO-2 (Fig 5D) had no significant change at 1.5 minute after SO_2_ derivatives injection compared with the vehicle group. PPG, BCA or NaHS pretreatment for 30 minutes also did not change the effect of SO_2_ on these indexes (Fig 5). These results suggest that NO and CO pathway might not be involved in the regulation of H_2_S on SO_2_ action.

## Discussion

Our study, for the first time, confirmed that the interaction between H_2_S and SO_2_ controls the basal blood pressure and vascular tone *in vivo* and *in vitro*. H_2_S negatively controls the plasma contents of SO_2_ and its vasorelaxant effect.

Both H_2_S and SO_2_ can be generated endogenously through the metabolic pathway of sulfur-containing amino acids in mammals [2,27–31]. Under certain biochemical conditions, H_2_S and SO_2_ can be converted to each other in cells. For example, neutrophils convert H_2_S to SO_2_ through oxidative stress [32]. H_2_S is oxidized by sulfide oxidase to generate thiosulfate, which is further converted to SO_2_ in the presence of thiosulfate sulfurtransferase [1]. In addition to metabolic pathways, crosstalk between these two gasotransmitters has also been observed in monocrotaline-induced pulmonary hypertensive rat model, where H_2_S was found to inhibit SO_2_ generation from lung tissues [18]. However, it was not clear whether there is a possible interaction between H_2_S and SO_2_ under physiological conditions. In this study, after the intravenous injection of PPG or BCA to inhibit endogenous H_2_S generation, rat plasma H_2_S levels decreased but SO_2_ levels increased. Conversely, injection of H_2_S donor NaHS to upregulate H_2_S levels could downregulate plasma SO_2_ contents. These results indicate that H_2_S inhibits the plasma levels of SO_2_, so as to maintain a low concentration of SO_2_ under physiological conditions. As for the mechanism by which H_2_S inhibits SO_2_ levels, a previous study showed that H_2_S could sulfhydrate AAT, a key enzyme catalyzing SO_2_ generation, to suppress its activity, thus inhibiting endogenous SO_2_ production and content [18].

Both H_2_S and SO_2_ are important gasotransmitters and play important roles in cardiovascular system [33,34]. Studies have shown that the administration of H_2_S or SO_2_ donors antagonizes hypertension in a variety of hypertensive animal models including spontaneously hypertensive rats and angiotensin II-induced hypertensive mice [35–37]. In the present study, MBP of rats was decreased rapidly after the intravenous injection of the SO_2_ donor (40 μmol/kg), and then returned to normal levels. This suggests that SO_2_ reduces blood pressure under physiological conditions, and this hypotensive effect is rapid and transient. However, the role of H_2_S in the regulation of basal blood pressure by SO_2_ is still unclear. In this study, after the intravenous injection of PPG or BCA to inhibit endogenous H_2_S production, the plasma H_2_S level was decreased, the SO_2_ level increased, and the hypotensive effect induced by SO_2_ was markedly promoted. Conversely, injection of NaHS to increase H_2_S level and decrease SO_2_ level could attenuate the hypotensive effect of SO_2_. These results suggest that the interaction between H_2_S and SO_2_ is important for the maintenance of physiological blood pressure.

It is known that the vasorelaxant effect of SO_2_ is one of the important mechanisms by which this compound exerts antihypertensive effect. The present study also showed that the treatment with SO_2_ donor could relax aortic rings. However, the role of H_2_S in the regulation of vascular tension by SO_2_ remains unclear. In this study, the pretreatment of aortic rings with PPG or BCA to suppress endogenous H_2_S generation could promote the vasorelaxation of SO_2_. While, pre-incubation of aortic rings with NaHS to increase H_2_S level could attenuate the vasorelaxation of SO_2_. These results suggest that and H_2_S negatively controls the vasorelaxant effect of SO_2_. Previous studies have reported that the K_ATP_ channel, calcium channel, and cGMP signaling are all involved in the vasorelaxant effect of H_2_S and SO_2_ [38–43]. These targets and signaling pathways might be responsible for the biological effects of sulfur-containing gasotransmitter networks. For example, both H_2_S and SO_2_ increase cGMP content. Although H_2_S does not directly activate soluble guanylate cyclase (sGC), it strongly inhibits phosphodiesterase (PDE) 5A to delay cGMP degradation [39]. SO_2_ not only promotes the formation of a heterodimer of sGC α and β subunits to activate sGC and promote cGMP synthesis but also inhibits PDE activity to suppress cGMP degradation, thus increasing the cGMP content [44]. The promotion of these two gasotransmitters at the cGMP level activates PKG signaling and induces vasodilatory effect. However, SO_2_ and H_2_S would not work alone, but rather H_2_S controls SO_2_ action, which might prevent excessive cGMP production. However, the possible comprehensive effects of the interaction between H_2_S and SO_2_ on these targets remain unknown, which merits further studies.

Previous studies showed that SO_2_ supplementation for 8 weeks upregulated plasma NO/eNOS pathway in the atherosclerotic rats [5], while H_2_S treatment for 2 h suppressed this pathway in the rat aortic tissues [45]. SO_2_ treatment for 24 h elevated the level of HO-1, producer of the gasotransmitter CO, in human skin keratinocytes [46], while blocking H_2_S production with PPG for 1 week induced HO-1 in rats [47]. In the present study, we found that that the plasma NO content and the expressions of aortic eNOS, HO-1 and HO-2 had no significant change at 1.5 minute after SO_2_ injection. PPG, BCA or NaHS pretreatment for 30 minutes also did not change the effect of SO_2_ on these indexes, suggesting that NO and CO pathway might not be involved in the regulation of H_2_S on SO_2_ action. Differences under experimental conditions might contribute to the discrepancy between our results and those reported in the literatures.

Although PPG is usually used as an inhibitor of H_2_S production, it is unspecific [48]. It also inhibits other pyridoxal phosphate (PLP) enzymes. In the present study, we also used another CSE inhibitor BCA to inhibit H_2_S production and observed that the effect of PPG could be reproduced. The potential off-target effects of these compounds must be kept in mind when interpreting the data. Therefore, genetic methods are needed to study the complex interaction between H_2_S and SO_2_ in the future studies.

The limitation of this study was that the effect of H_2_S on the SO_2_-induced vasodilation was measured in the aorta. Although the change of vascular reactivity of this conductance vessel could be used as an early indicator of the development of diseases related to abnormal blood pressure, the resistance artery is more closely correlated to blood pressure control. Therefore, it is necessary to further confirm the effect of the interaction between H_2_S and SO_2_ on vasodilation in arteries such as mesenteric arteries in the future. Factors such as pH, trace metals and oxygen content in buffers, material of measuring container, oxidant of sulfide and reaction of sulfide with many different species (including superoxide radical, hydrogen peroxide and peroxynitrite) were found to affect the determination of H_2_S, resulting in the highly variable absolute H_2_S concentration in biological samples measured by different methods [49]. In fact, measuring H_2_S concentrations is still a matter of debate. There is no unified method for the determination of H_2_S in plasma or tissue samples. Some studies show that plasma sulfide is well below 1 μM, and still many studies indicate that the plasma sulfide levels determined by different methods including liquid chromatography-mass spectrometry, methylene blue method, H_2_S-selective sensor and H_2_S-specific fluorescent probes were tens of micromoles [21,49–56], which was consistent with our results. In the future, highly selective, sensitive and accurate H_2_S measurement methods are still needed to improve the unresolved limitations of the current methods.

## Conclusion

**Fig 6:**
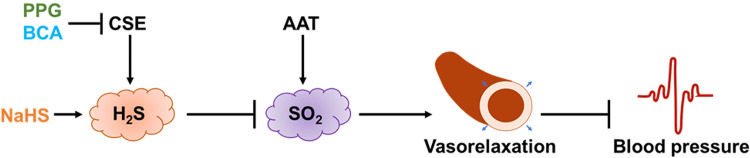
H_2_S negatively controls the plasma content of SO_2_, its vasorelaxant effect and transient hypotensive response.

Our *in vivo* and *in vitro* findings provide new clues for the integrated regulation of H_2_S and SO_2_ to control blood pressure and vascular tension. Our results suggest that H_2_S negatively controls the plasma content of SO_2_, the vasorelaxant effect and the transient hypotensive response (Fig 6). The findings indicate that SO_2_ acts as a compensatory defense gaseous molecule for H_2_S when it is disturbed, playing a vascular protective role. A better understanding of the integrated regulation of these two sulfur-containing gasotransmitters in vascular function would provide new ideas to further explore the pathogenesis of cardiovascular diseases and reveal new targets for their treatment in the future.

## Supporting information

S1 Fig The interconversion of SO_3_^2–^, HSO_3_^–^ and SO_2_. (TIF)

S2 Fig The effects of pre-administrated PPG, BCA or NaHS on rat blood pressure. Changes in mean blood pressure of rats within 1 minute after injected with PPG (30 mg/kg), BCA (50 mg/kg) or NaHS (56 μmol/kg). Data are mean±SEM; n = 8 in each group; Student’s t-test; *P<0.05 compared with zero time point.
(TIF)

S1 Data Raw data. (RAR)
