# Supplementary figures and images for: Role of hydrogen sulfide in sulfur dioxide production and vascular regulation
Source: PLoS One. 2022 Mar 17;17(3):e0264891. doi: 10.1371/journal.pone.0264891 (PMC8929647; doi:10.1371/journal.pone.0264891)

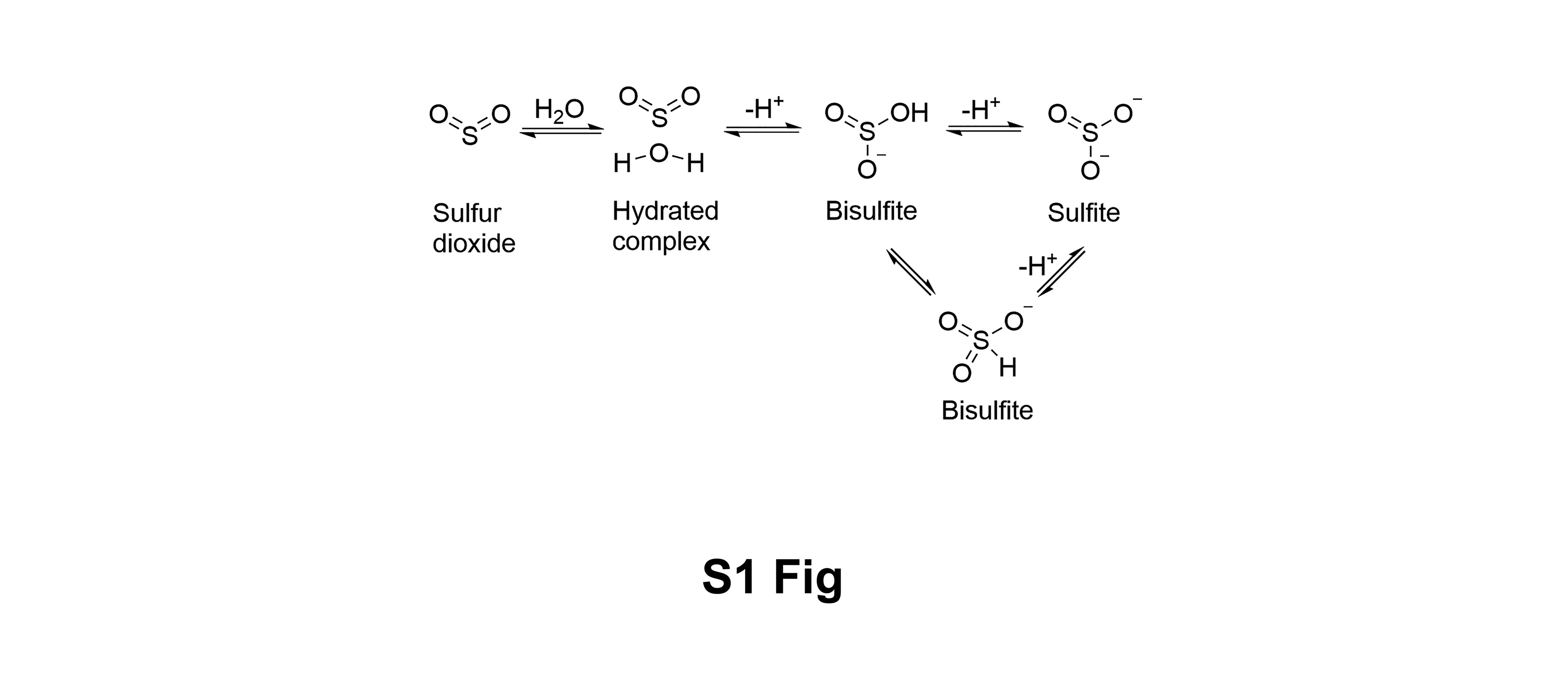

Supplement: S1 Fig — (TIF) [file pone.0264891.s001.tif]

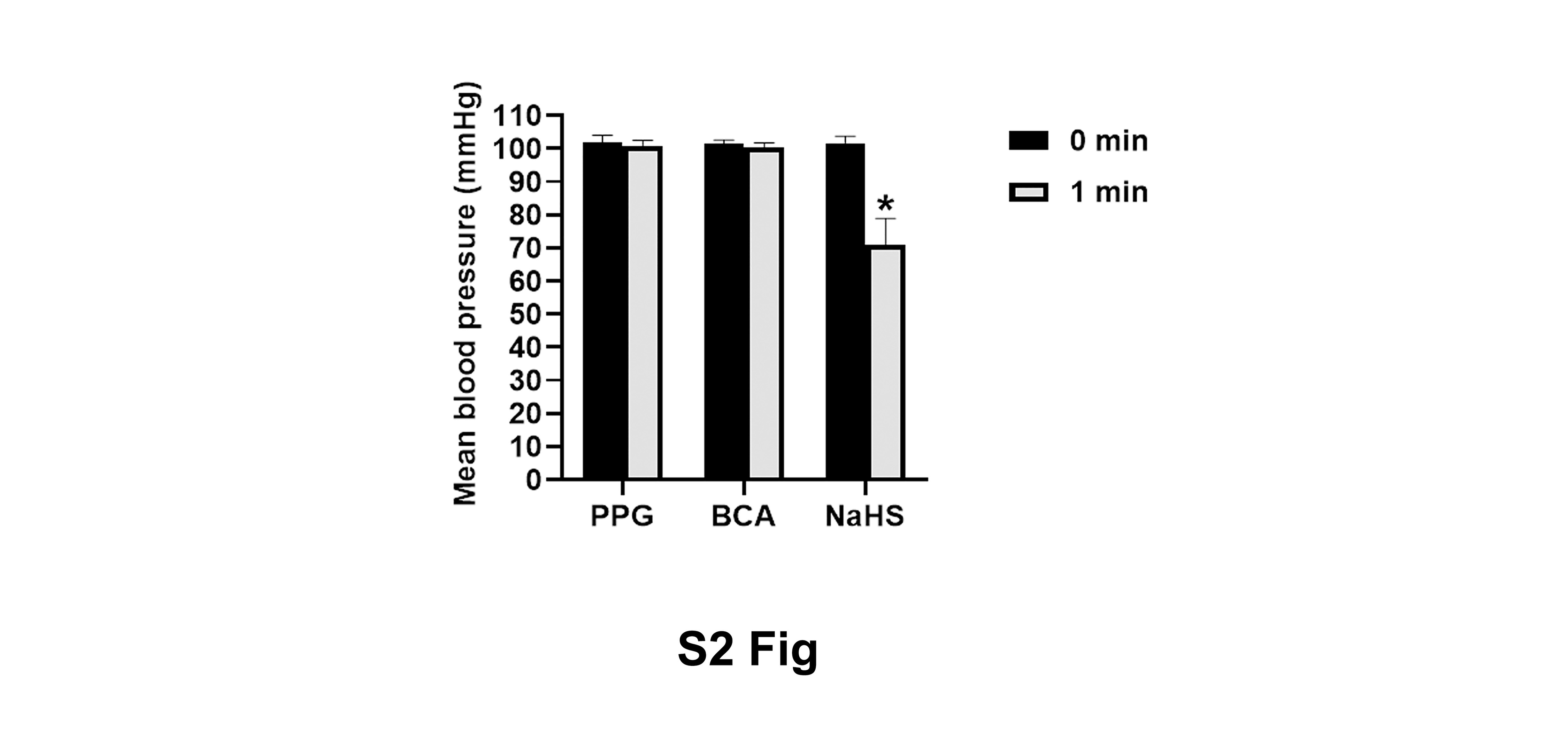

Supplement: S2 Fig — Changes in mean blood pressure of rats within 1 minute after injected with PPG (30 mg/kg), BCA (50 mg/kg) or NaHS (56 μmol/kg). Data are mean±SEM; n = 8 in each group; Student’s t-test; *P<0.05 compared with zero time point. (TIF) [file pone.0264891.s002.tif]
